# Palliative primary tumor resection provides survival benefits for the patients with metastatic colorectal cancer and low circulating levels of dehydrogenase and carcinoembryonic antigen

**DOI:** 10.1186/s40880-016-0120-4

**Published:** 2016-06-29

**Authors:** Wen-Zhuo He, Yu-Ming Rong, Chang Jiang, Fang-Xin Liao, Chen-Xi Yin, Gui-Fang Guo, Hui-Juan Qiu, Bei Zhang, Liang-Ping Xia

**Affiliations:** State Key Laboratory of Oncology in South China, Collaborative Innovation Center for Cancer Medicine, Sun Yat-sen University Cancer Center, Guangzhou, 510060 Guangdong P. R. China; VIP Region, Sun Yat-sen University Cancer Center, Guangzhou, 510060 Guangdong P. R. China

**Keywords:** Metastatic colorectal cancer, Palliative primary tumor resection, Overall survival, Lactate dehydrogenase, Carcinoembryonic antigen

## Abstract

**Background:**

It remains controversial whether palliative primary tumor resection (PPTR) can provide survival benefits to the patients with metastatic colorectal cancer (mCRC) who have unresectable metastases. The aim of this study was to evaluate whether PPTR could improve the survival of patients with mCRC.

**Methods:**

We conducted a retrospective study on consecutive mCRC patients with unresectable metastases who were diagnosed at Sun Yat-sen University Cancer Center in Guangzhou, Guangdong, China, between January 2005 and December 2012. Overall survival (OS) and progression-free survival (PFS) after first-line chemotherapy failure were compared between the PPTR and non-PPTR patient groups.

**Results:**

A total of 387 patients were identified, including 254 who underwent PPTR and 133 who did not. The median OS of the PPTR and non-PPTR groups was 20.8 and 14.8 months (*P* < 0.001), respectively. The median PFS after first-line chemotherapy was 7.3 and 4.8 months (*P* < 0.001) in the PPTR and non-PPTR groups, respectively. A larger proportion of patients in the PPTR group (219 of 254, 86.2%) showed local progression compared with that of patients in the non-PPTR group (95 of 133, 71.4%; *P* < 0.001). Only patients with normal lactate dehydrogenase (LDH) levels and with carcinoembryonic antigen (CEA) levels <70 ng/mL benefited from PPTR (median OS, 22.2 months for the PPTR group and 16.2 months for the non-PPTR group; *P* < 0.001).

**Conclusions:**

For mCRC patients with unresectable metastases, PPTR can improve OS and PFS after first-line chemotherapy and decrease the incidence of new organ involvement. However, PPTR should be recommended only for patients with normal LDH levels and with CEA levels <70 ng/mL.

## Background

Approximately 25% of patients with colorectal cancer present with synchronous metastases at the time of diagnosis [1, 2]. Although curative surgery, including eradication of both the primary cancer and the metastatic lesions, can be successful in patients with limited metastasis, most patients remain incurable owing to unresectable metastases [3]. For such patients, both the National Comprehensive Cancer Network and the European Society for Medical Oncology recommend chemotherapy without primary cancer resection except in cases of intestinal perforation, intestinal obstruction, or other emergencies [4]. However, increasing evidence indicates that palliative primary tumor resection (PPTR) plus chemotherapy could extend patients’ survival [5–7], as shown in Table 1, which challenges these recommendations.

**Table 1:** Summary of studies that evaluated the role of PPTR

First author and reference number	Period of patient involvement	Chemotherapy regimen	PPTR	No. of patients	Median OS (months)	*P*
Seo [4]	2001–2008	Oxaliplatin, irinotecan, and 5-FU	Yes	144	22	0.076
			No	83	14	
Sabine [16]	2003–2004	Irinotecan, oxaliplatin, and capecitabine	Yes	258	16.7	<0.001
			No	141	11.4	
Sabine [16]	2005–2006	Oxaliplatin and capecitabine	Yes	289	20.7	<0.001
			No	159	13.4	
Leyo [17]	1996–1999	Unknown	Yes	127	16	<0.001
			No	103	9	
Ferrand [18]	1997–2001	5-FU + CF, 5-FU, or raltitrexed	Yes	156	16.3	<0.001
			No	60	9.6	
Mehdi [19]	1998–2007	FOLFOX, FOLFIRI, XELOX, or 5-FU	Yes	85	30.7	0.031
			No	123	21.9	
Tebutt [20]	1990–1999	5-FU, raltitrexed, capecitabine, or uracil tegafur	Yes	280	14	0.080
			No	82	8.2	
Martyn [21]	1999–2006	Unknown	Yes	45	11	0.206
			No	52	7	

*PPTR* palliative primary tumor resection, *OS* overall survival, *5-FU* 5-fluorouracil, *CF* cisplatin and 5-fluorouracil, *FOLFOX* folinic acid 5-fluorouracil and oxaliplatin, *FOLFIRI* folinic acid, 5-fluorouracil, and irinotecan, *XELOX* xeloda and oxaliplatin

The specific benefits of PPTR remain undefined, because all the published studies are retrospective and have reported contradictory results; no randomized controlled studies have been reported. In addition, in a previous study, the effect of palliative surgery on survival was confounded by the differential use of chemotherapy drugs [8]. Furthermore, some studies did not provide details about the chemotherapy used [9–11]. Other studies included patients who were treated between 1980 and 2000 [9]; during this period, chemotherapy for metastatic colorectal cancer (mCRC) changed from 5-fluorouracil-based two-agent regimens to oxaliplatin- or irinotecan-based three-agent regimens. Similarly, the principles underlying the management of metastatic lesions also changed during this period. Also, the reliability of comparing one study with another is inevitably challenged by patient selection bias.

Apart from the ongoing randomized clinical trials, an alternative way to resolve the question is to identify a method or index for selecting patients who are likely to benefit from PPTR. To date, only one study has indicated that patients with rectal cancer or with low levels of carcinoembryonic antigen (CEA) are likely to benefit from PPTR [12]. Accordingly, in the age of modern chemotherapy, we investigated the effect of PPTR on the survival of patients with synchronous mCRC. Additionally, we determined which patients are likely to benefit from PPTR.

## Methods

### Patient selection

We reviewed the database from Sun Yat-sen University Cancer Center and selected patients who met the following criteria: (1) between 2005 and 2012, they were diagnosed with mCRC at first diagnosis; (2) they had an Eastern Cooperative Oncology Group status ≤2; and (3) their follow-up information was available. The following patients were excluded: (1) those who had evidence of intestinal obstruction, enterobiasis, or bleeding at the time of first presentation; (2) those who had a second primary tumor; and (3) those who had all visible tumors removed. Informed consent was obtained from all patients involved in this study, which was approved by the ethical committee of Sun Yat-sen University Cancer Center.

### Detection of CEA

CEA level was evaluated using electrochemiluminescence with the Roche Elecsys 2010 Chemistry Analyzer (Basel, Switzerland).

### Patient follow-up and statistical analysis

Overall survival (OS) was defined as the time from diagnosis to death or the last follow-up; progression-free survival (PFS) after first-line chemotherapy was defined as the time from the initiation of first-line chemotherapy to disease progression or death. The calculation date was October 31, 2014.

SPSS version 13.0 software (Statistical Product and Service Solutions, Chicago, IL, USA) was used to perform statistical analyses. The Kaplan–Meier method was used to plot the survival curves, and the differences were compared using the log-rank test. Multivariate analysis and the Cox proportional hazards model were used to determine independent significance. A *P* value less than or equal to 0.05 was considered significant.

## Results

### Patient characteristics

**Fig. 1:**
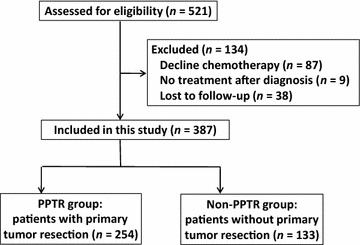
Flow chart of the inclusion of patients with metastatic colorectal cancer. *PPTR* palliative primary tumor resection

In our database, we identified 521 mCRC patients with synchronous metastases. Of these patients, 87 underwent surgery without chemotherapy, 9 did not receive any treatment after diagnosis, and 38 were lost to follow-up; the remaining 387 patients were included in our analysis (Fig. 1). The patients’ clinicopathologic characteristics at baseline are listed in Table 2.

**Table 2:** Characteristics of the 387 colorectal cancer patients with unresectable metastasis

Characteristic	Variable	Total	PPTR group	Non-PPTR group	*P*
Age (years)	≤60	210	135	75	0.543
	>60	177	119	58	
Sex	Men	259	169	90	0.822
	Women	128	85	43	
Location	Right	132	87	45	0.120
	Left	124	89	35	
	Rectal	131	78	53	
First-line chemotherapy	Oxaliplatin-based	335	221	114	0.723
	Irinotecan-based	52	33	19	
Metastasis organs	1	283	194	89	0.108
	2	92	52	40	
	≥3	12	8	4	
CEA (ng/mL)	≤5	81	52	29	0.760
	>5	306	202	104	
ALP (U/L)	≤110	315	207	108	0.944
	>110	72	47	25	
LDH (U/L)	≤245	257	192	65	<0.001
	>245	130	62	68	

All values are presented as number of patients

*PPTR* palliative primary tumor resection, *CEA* carcinoembryonic antigen, *ALP* alkaline phosphatase, *LDH* lactate dehydrogenase

Among the 387 patients studied, 109 refused surgery, 254 underwent PPTR, and 24 underwent an exploratory celiotomy. Of the patients who underwent a celiotomy, 16 had their abdomen closed immediately because peritoneal metastases and other organ involvement were found; the remaining eight patients underwent a prophylactic enterostomy because of the advanced stage of the primary tumor. Both the patients who refused surgery and those who underwent an exploratory celiotomy were included in the non-PPTR group.

None of the 254 patients who underwent PPTR died as a result of surgery. The median interval between PPTR and systemic chemotherapy was 24 days (range, 13–58 days). Of these patients, 221 underwent PPTR followed by chemotherapy, and 33 underwent chemotherapy followed by PPTR. All 133 patients in the non-PPTR group accepted palliative chemotherapy, either oxaliplatin-based or irinotecan-based.

### The effect of PPTR on OS

Patients who underwent PPTR had a significantly longer OS than patients who did not (median: 20.8 vs. 14.8 months, *P* < 0.001). We also evaluated the prognostic values of all the factors, including age, sex, primary tumor location, metastatic site, regimen of first-line chemotherapy, and alkaline phosphatase (ALP), lactate dehydrogenase (LDH), and CEA levels (Table 2). ALP (*P* < 0.001), LDH (*P* < 0.001), and CEA (*P* < 0.001) levels were all prognostic factors. As shown in Table 3, a multivariate analysis including PPTR as well as ALP, LDH, and CEA levels indicated that PPTR (*P* = 0.009) and LDH level (*P* = 0.011) were independent prognostic factors.

**Table 3:** Multivariate analysis of prognostic factors in 387 patients with metastatic colorectal cancer

Variable	B	*P*	Exp(B)	95% CI for exp(B)
PPTR	−0.452	0.009	0.636	0.453–0.893
ALP	−0.210	0.295	0.810	0.546–1.201
LDH	−0.453	0.011	0.636	0.450–0.900
CEA	−0.161	0.520	0.851	0.522–1.390

*PPTR* palliative primary tumor resection, *CEA* carcinoembryonic antigen, *ALP* alkaline phosphatase, *LDH* lactate dehydrogenase, *CI* confidence interval

Next, we studied the distribution of clinicopathologic characteristics at baseline in the PPTR group and non-PPTR group patients (Table 2). Elevated LDH level was observed in 51.1% (68 of 133) of patients in the non-PPTR group, but only 24.4% (62 of 254) of patients in the PPTR group had high LDH level (*P* < 0.001). The other factors, including age, sex, primary tumor location, metastatic site, regimen of first-line chemotherapy, and ALP and CEA levels, were similar between the two groups.

### The effect of PPTR followed by first-line chemotherapy on PFS

The median PFS after first-line chemotherapy of patients in the PPTR and non-PPTR groups were 7.3 and 4.8 months, respectively (*P* < 0.001). Among the factors listed in Table 2, ALP (*P* < 0.001), LDH (*P* < 0.001), and CEA (*P* < 0.001) levels could all be used to distinguish patients with different PFS after the first-line chemotherapy. A multivariate analysis including PPTR and ALP, LDH, and CEA levels indicated that PPTR (*P* = 0.014) and LDH level (*P* = 0.036) were independent factors.

### The effect of PPTR on first-line chemotherapy failure

In this study, all patients accepted first-line chemotherapy; each of them accepted at least four cycles of chemotherapy. We classified the patients into two groups according to the nature of first-line chemotherapy failure. If new organs were involved in the progression, it was defined as systematic progression; if only the primary tumor was enlarged or new lesions were emerged only in organs that had already been involved, it was defined as local progression. We found local progression in a larger proportion of PPTR group patients compared with non-PPTR group patients (86.2% [219/254] vs. 71.4% [95/133], *P* < 0.001).

### LDH and CEA levels could identify patients who may benefit from PPTR

Since LDH distribution was not balanced between the PPTR and non-PPTR groups, we classified and analyzed patient survival according to LDH level. Normal LDH level was observed in 257 patients; of these patients, 192 underwent PPTR, and 65 did not. The OS of the PPTR group and non-PPTR group patients with normal LDH level was 22.4 and 15.6 months, respectively (*P* < 0.001). The PFS after first-line chemotherapy of the PPTR group and non-PPTR group patients who had normal LDH levels was 7.8 and 5.5 months, respectively (*P* < 0.001). Other factors were similar between the two groups, as shown in Table 4. Elevated LDH level was observed in 130 patients; of these patients, 62 accepted PPTR, and 68 did not. The OS of the PPTR group and non-PPTR group patients with elevated LDH level was 18.9 and 12.9 months, respectively (*P* = 0.268). The PFS after first-line chemotherapy of the PPTR group and non-PPTR group patients with elevated LDH level was 5.6 and 4.7 months, respectively (*P* = 0.100).

**Table 4:** Characteristics of the 257 colorectal cancer patients with unresectable metastasis who had normal LDH levels

Characteristic	Variable	Total	PPTR group	Non-PPTR group	*P*
Age (years)	≤60	141	103	38	0.500
	>60	116	89	27	
Sex	Men	180	134	46	0.882
	Women	77	58	19	
Location	Right	98	75	23	0.799
	Left	80	60	20	
	Rectal	79	57	22	
First-line chemotherapy	Oxaliplatin-based	228	171	57	0.763
	Irinotecan-based	29	21	8	
Metastasis organs	1	201	152	49	0.733
	2	51	36	15	
	≥3	5	4	1	
CEA (ng/mL)	≤5	62	45	17	0.658
	>5	195	147	48	
ALP (U/L)	≤110	223	166	57	0.800
	>110	34	26	8	

All values are presented as number of patients

*PPTR* palliative primary tumor resection, *CEA* carcinoembryonic antigen, *ALP* alkaline phosphatase, *LDH* lactate dehydrogenase

Since a wide range in the degree of LDH elevation was observed, we divided the patients with increased LDH levels into two groups (upper and lower halves) according to the degree of elevation to identify patients who could potentially benefit from PPTR. As shown in Table 5, we found that neither group benefited from PPTR.

**Table 5:** Overall survival of the patients grouped by LDH levels

LDH level (U/mL)	Overall survival (months)		*P*
	PPTR group	Non-PPTR group	
≤245	22.43 (4.07–86.73)	15.63 (2.80–64.50)	<0.001
246–380	19.50 (4.70–65.37)	12.23 (2.33–44.43)	0.243
>380	16.83 (2.77–73.20)	15.37 (3.23–41.33)	0.892

Each value is presented as median followed by range in parentheses

The overall survival was compared between PPTR and non-PPTR group, and the differences were analyzed using the log-rank test

*LDH* lactate dehydrogenase, *PPTR* palliative primary tumor resection

In addition to LDH level, ALP and CEA levels—the other significant prognostic factors demonstrated in our study—were investigated to determine which patients might benefit from PPTR. Normal ALP level was observed in 305 patients; of these patients, 221 accepted PPTR, and 84 did not. The OS of the PPTR group and non-PPTR group patients with normal ALP levels was 20.9 months (range, 2.8–86.7 months) and 16.3 months (range, 3.2–64.5 months), respectively (*P* = 0.001). Increased ALP levels were observed in 82 patients; of these patients, 33 accepted PPTR, and 49 did not. The OS of the PPTR group and non-PPTR group patients with elevated ALP levels was 19.5 months (range, 4.7–34.9 months) and 11.3 months (range, 2.3–42.3 months), respectively (*P* = 0.035). Because patients with both increased ALP levels and normal ALP levels could benefit from PPTR, ALP was not a predictor of survival benefit. Previous studies have suggested that patients with CEA levels >600 ng/mL did not benefit from PPTR [12]. In our study, 32 patients had CEA levels >600 ng/mL. Of these patients, 11 accepted PPTR, and 21 did not. The OS of the PPTR group and non-PPTR group patients with CEA levels >600 ng/mL was 17.1 months (range, 8.4–26.0 months) and 12.9 months (range, 7.2–29.8 months), respectively (*P* = 0.582). However, since only 8.3% (32 of 387) of the patients had CEA levels >600 ng/mL, the clinical significance is limited. We divided the patients with increased CEA levels into three groups: patients in the upper third were classified into group A, patients in the middle third were classified into group B, and patients in the lower third were classified into group C; patients with normal CEA levels were classified into group D. As shown in Table 6, we found that patients did not benefit from PPTR if they had CEA levels >70 ng/mL.

**Table 6:** Overall survival of the patients with colorectal cancer grouped by CEA levels

CEA level (ng/mL)	Overall survival (months)		*P*
	PPTR group	Non-PPTR group	
0–5	26.13 (2.80–86.73)	16.83 (2.80–64.50)	0.032
5.1–18	20.90 (2.77–70.30)	14.80 (5.73–41.33)	0.011
18.1–70	19.70 (2.87–63.17)	11.85 (2.33–36.63)	0.002
>70	19.57 (4.70–73.33)	14.17 (3.47–44.43)	0.186

Each value is presented as median followed by range in parentheses

The overall survival was compared between PPTR and non-PPTR group, and the differences were analyzed using the log-rank test

*CEA* carcinoembryonic antigen, *PPTR* palliative primary tumor resection

**Fig. 2:**
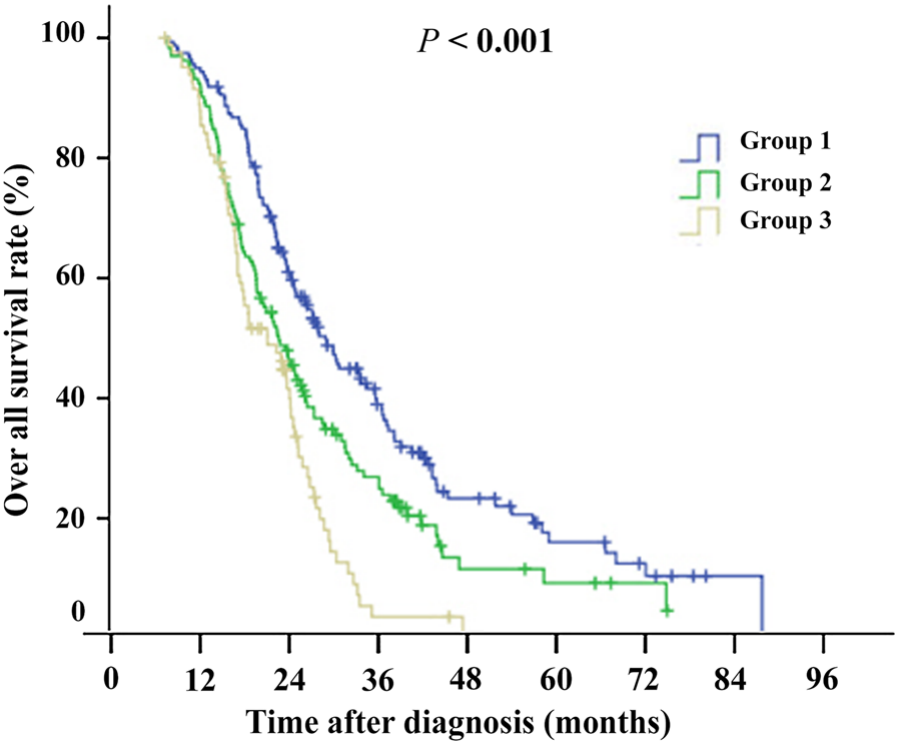
Overall survival of 387 patients with metastatic colorectal cancer grouped by lactate dehydrogenase (LDH) and carcinoembryonic antigen (CEA) levels. *Group 1* patients who had normal LDH levels and CEA levels ≤70 ng/mL; *group 2* patients with increased LDH levels and CEA levels ≤70 ng/mL, or patients with normal LDH levels and CEA levels >70 ng/mL; *group 3* patients who had increased LDH levels and CEA levels >70 ng/mL

Since both LDH and CEA levels showed the potential to be used for selecting patients who could benefit from PPTR, we investigated whether a combination of the two indexes could provide a better predictive model. We classified the patients into three groups. Group 1 consisted of patients who had normal LDH levels and CEA levels ≤70 ng/mL; group 2 consisted of patients with increased LDH levels and CEA levels ≤70 ng/mL, or patients with normal LDH levels and CEA levels >70 ng/mL; and group 3 consisted of patients who had increased LDH levels and CEA levels >70 ng/mL. As shown in Fig. 2, the OS of groups 1, 2, and 3 were 20.8 months (range, 2.8–86.7 months), 17.4 months (range, 2.3–73.3 months) and 15.7 months (range, 3.5–44.4 months), respectively (*P* < 0.001). We also compared the OS of the PPTR group and non-PPTR group patients in each group. As shown in Table 7, PPTR patients in group 1 had a significantly longer OS than non-PPTR patients in group 1 (22.2 vs. 16.2 months, *P* < 0.001).

**Table 7:** Overall survival of the patients grouped by the combination of LDH and CEA levels

Group	PPTR group	Non-PPTR group	*P*
Group 1	22.23 (4.07–86.73)	16.23 (2.80–64.50)	<0.001
Group 2	20.13 (2.77–73.33)	13.17 (2.33–41.33)	0.188
Group 3	18.50 (4.70–25.50)	13.55 (3.47–44.43)	0.918

Each value is presented as median followed by range in parentheses

Group 1 consisted of patients who had normal LDH levels and CEA levels ≤70 ng/mL, group 2 consisted of patients with increased LDH levels and CEA levels ≤ 70 ng/mL, or patients with normal LDH levels and CEA levels >70 ng/mL, group 3 consisted of patients who had increased LDH levels and CEA levels >70 ng/mL. The overall survival was compared between PPTR and non-PPTR group, and the differences were analyzed using the log-rank test

*PPTR* palliative primary tumor resection, *CEA* carcinoembryonic antigen, *LDH* lactate dehydrogenase

## Discussion

In the present study, we analyzed the role of PPTR from the following aspects: its effect on intestinal complications, its effect on patient survival, and its safety. We found that only patients with normal LDH levels and CEA levels <70 ng/mL gained survival benefits from PPTR.

The first potential advantage of PPTR is to reduce the incidence of potential intestinal complications during chemotherapy. However, the incidence of death and major complications, including those associated with surgical intervention, was reported to be 14% among patients treated with folinic acid (or leucovorin), 5-fluorouracil, and oxaliplatin, a combination better known as FOLFOX6, plus bevacizumab [13]. Reports from Memorial Sloan Kettering Cancer Center [14] and Fox Chase Cancer Center [15] showed that only 7% and 9.8% of patients, respectively, needed surgical intervention for intestinal complications experienced during chemotherapy. Furthermore, a meta-analysis that included seven studies involving 850 patients showed that the incidences of intestinal obstruction and hemorrhage were 13.9% and 3.0%, respectively [1]. Thus, these data suggest that prophylactic primary cancer resection before chemotherapy is not necessary.

The second indication for PPTR is that it may extend patient survival. However, these survival benefits are controversial, and previous studies have had several drawbacks, including unknown or outdated chemotherapy regimens and patient selection biases. The patients in our study were diagnosed after 2005 and were given either the standard mFOLFOX6 regimen (folinic acid, 5-fluorouracil, and oxaliplatin) or FOLFIRI (folinic acid, 5-fluorouracil, and irinotecan) as first-line chemotherapy. Efforts were also made to limit patient selection bias. First, we compared the age distribution, performance status score, tumor location, and metastasis site between the PPTR and non-PPTR groups. Second, the distribution of the potential prognostic factors, including ALP, LDH, and CEA levels, was also compared. All the mentioned indexes except LDH levels were balanced between the two groups. We found that PFS after first-line chemotherapy was significantly extended in the PPTR group, which could be important in understanding the advantage of PPTR; we do not think that patient selection bias influenced this PFS. In addition, chemotherapy discontinuation did not occur in our study, which suggests that patients in both groups tolerated chemotherapy well. However, the mechanism of how PPTR can extend PFS remains unclear.

Although the indications for PPTR are not established, short life expectancy, terminal stage, and poor general patient condition are usually considered contraindications for surgery. To determine which patients can benefit from PPTR, we investigated whether the three prognostic factors found in our study—ALP, LDH, and CEA levels—could identify these patient populations. First, we found that only patients with normal LDH could get survival benefit from PPTR. We further classified patients with elevated LDH levels into the upper and lower halves and confirmed that neither benefited from PPTR. A one-pool analysis reported at the American Society of Clinical Oncology annual meeting in 2012 indicated that patients with low CEA levels can benefit from PPTR [12], but the benefit was lost once CEA levels increased to greater than 600 ng/mL. We found similar results; however, the cutoff of CEA level in our study was 70 ng/mL. Finally, the combination of LDH and CEA levels was a better predictor of patient benefit than either LDH or CEA levels alone. This superiority was reflected in three aspects. The first aspect is that the combination of the two indexes can be used to select patients in the normal LDH group with relatively poor prognosis and patients in the elevated LDH group with relatively good prognosis; both of these were defined as moderate-risk groups, as shown in Fig. 1. The second aspect is that patients in the moderate-risk groups do not benefit from PPTR. The third and most important aspect is that only patients with a very good prognosis, as opposed to those with a moderate or poor prognosis, benefit from PPTR.

The safety of PPTR was another unresolved question. No surgery-related deaths were found in our study. The average interval between chemotherapy and surgery was 24 days, which is very similar to the 3-week interval usually required for adjuvant chemotherapy. However, a recent meta-analysis found 2.7% of postoperative mortality rate and 11.8% of overall serious complications [1], indicating that PPTR should only be undertaken with caution.

Our study had several limitations. First, the retrospective nature limited its power. Second, we did not further divide LDH into subtypes, such as LDH-1 and LDH-5. Third, some elderly patients might have had cardiovascular disease, which could also affect LDH levels. Further prospective studies are needed to validate the results of our study.

Although the distributions of CEA were balanced between the PPTR group and non-PPTR group, it is noteworthy that there were significant metastatic organ differences between the PPTR and non-PPTR groups in our study. The unbalanced distribution of metastasis organs may reflect the fact that patients with a smaller tumor burden are more likely to implement PPTR in clinical practice. We acknowledge that this is a potential weakness of our study. We think that close attention should be paid to tumor burden and metastasis organs when future investigations are designed.

In conclusion, we found that PPTR could extend OS and PFS after first-line chemotherapy and decrease the incidence of new organ involvement in patients with mCRC who had unresectable metastases. Although no serious surgery-related complications occurred, PPTR should be recommended only for patients with normal LDH levels and CEA levels <70 ng/mL. However, further validation studies are needed.
